# Humidity Sensors Printed on Recycled Paper and Cardboard

**DOI:** 10.3390/s140813628

**Published:** 2014-07-28

**Authors:** Matija Mraović, Tadeja Muck, Matej Pivar, Janez Trontelj, Anton Pleteršek

**Affiliations:** 1 Pulp and Paper Institute, Bogišićeva 8, 1000 Ljubljana, Slovenia; E-Mail: matija.mraovic@icp-lj.si; 2 Chair of Information and Graphic Arts Technology, Faculty of Natural Sciences and Engineering, University of Ljubljana, Snežniška ulica 5, 1000 Ljubljana, Slovenia; E-Mails: tadeja.muck@gmail.com (T.M.); matej.pivar@gmail.com (M.P.); 3 Faculty of Electrical Enginnering, University of Ljubljana, Trzaska 25, 1000 Ljubljana, Slovenia; E-Mail: janez.trontelj1@guest.arnes.si; 4 ams AG (ams R&D), Tehnološki Park 21, 1000 Ljubljana, Slovenia

**Keywords:** printed electronics, pulp, screen-printed sensors, smart labels, radio frequency identification (RFID), passive tag, UHF data logger SL900A

## Abstract

Research, design, fabrication and results of various screen printed capacitive humidity sensors is presented in this paper. Two types of capacitive humidity sensors have been designed and fabricated via screen printing on recycled paper and cardboard, obtained from the regional paper and cardboard industry. As printing ink, commercially available silver nanoparticle-based conductive ink was used. A considerable amount of work has been devoted to the humidity measurement methods using paper as a dielectric material. Performances of different structures have been tested in a humidity chamber. Relative humidity in the chamber was varied in the range of 35%–80% relative humidity (RH) at a constant temperature of 23 °C. Parameters of interest were capacitance and conductance of each sensor material, as well as long term behaviour. Process reversibility has also been considered. The results obtained show a mainly logarithmic response of the paper sensors, with the only exception being cardboard-based sensors. Recycled paper-based sensors exhibit a change in value of three orders of magnitude, whereas cardboard-based sensors have a change in value of few 10s over the entire scope of relative humidity range (RH 35%–90%). Two different types of capacitor sensors have been investigated: lateral (comb) type sensors and modified, perforated flat plate type sensors. The objective of the present work was to identify the most important factors affecting the material performances with humidity, and to contribute to the development of a sensor system supported with a Radio Frequency Identification (RFID) chip directly on the material, for use in smart packaging applications. Therefore, the authors built a passive and a battery-supported wireless module based on SL900A smart sensory tag's IC to achieve UHF-RFID functionality with data logging capability.

## 1. Introduction

Printed electronics using electrically functional inks and traditional printing technologies (flexo, screen, offset and gravure) are set to revolutionize the fabrication of various electronic devices on flexible substrate materials such as plastic foils, paper and textiles. Research, design, fabrication and results of various screen printed capacitive humidity sensors was presented in [1]. The possibility of printing UHF RFID antennas on various low-cost and low-quality materials was analyzed in [2]. Some research was also done on electronics printed on recycled papers [3,4]. Many studies have reported various applications in printing electronic devices such as sensors, strain gauges, displays and radio frequency identification (RFID) tags [5–8].

The development of sensors for low cost and accurate humidity measurements is essential for applications in the environmental, agriculture, medical and semiconductor industries [9,10]. In the transport, construction industry and society high humidity conditions are in general the major cause of material failure and material fatigue. Printed electronics provide a means for low cost manufacture of humidity sensor systems, which can be directly integrated into the materials [11].

Humidity sensors can be of different types such as capacitive type, resistive, colorimetric, or surface acoustic wave (SAW), based on the sensing principle [12–15]. Capacitive type sensors are linear, require less complex circuitry and can operate over a wide range of humidity [16].

Many studies have reported printed capacitive humidity type sensors. A humidity sensor that employs interdigitated capacitors (IDC) printed with silver (Ag) nanoparticle based ink on a flexible polyethylene terephthalate (PET) substrate was successfully fabricated using a gravure printing process [17]. Small, low-cost and flexible humidity sensors were designed and fabricated by printing silver electrodes on a thin polyimide film using an inkjet-printing [18].

Other research was done using screen-printing and dry-phase patterning. A wireless label for humidity detection was successfully manufactured. It is made up of a printed antenna for wireless communication coupled with a printed capacitor (sensor head) for humidity detection. Changes in humidity level are detected as a shift in the resonant frequency of a label due to different humidity conditions and change in the sensor capacitance [11].

In presented study our approach was to develop electric capacitive sensors for humidity sensing of various configurations and geometries via the screen printing method with silver based conductive ink on different cellulose substrates such as recycled paper and cardboard and a few synthetic materials such as poly-carbonate and PET foil.

## 2. Experimental Section

**Figure 1.**
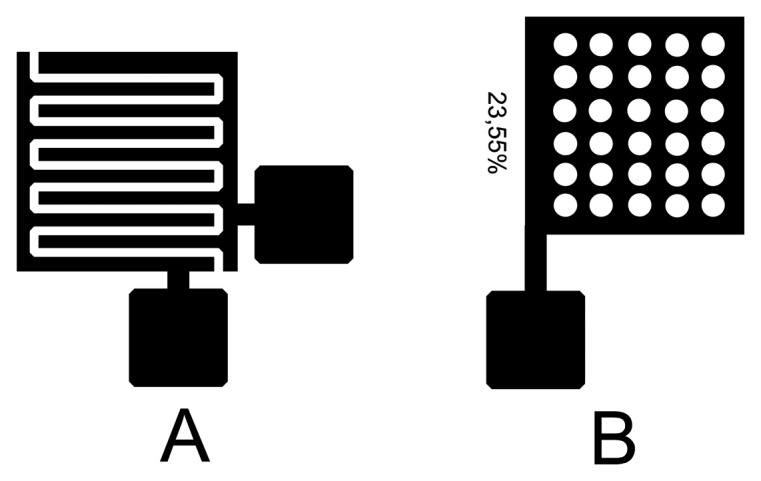
2nd generation sensors (**A**—lateral capacitor, **B**—perforated flat plate capacitor).

Research work started with fabrication of simple flat plate capacitors, where flat solid plate electrodes were screen printed with conductive ink on both sides of the substrate (paper, cardboard), thus the substrate functioned as the dielectric layer in a flat plate capacitor. The results obtained from first generation sensors served as an orientation and concept analysis for the development of second generation sensor designs—improved flat plate capacitors and comb type sensors. To improve responses of the flat plate type sensors perforated electrodes were used instead of solid ones (Figure 1).

### 2.1. Design of Sensors

**Figure 2.**
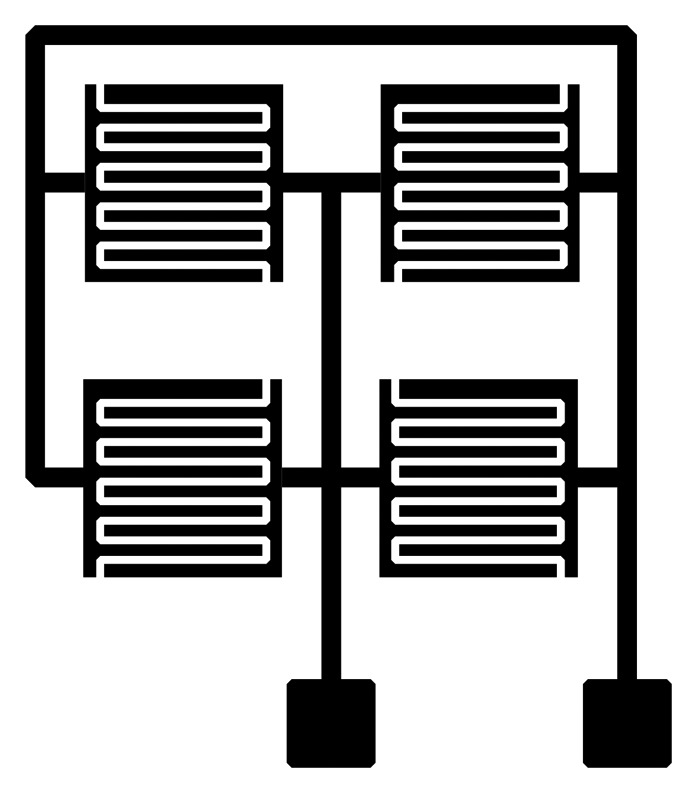
Lateral sensor array.

Sensors have been designed in three sizes: 1 × 1, 2 × 2 and 3 × 3 cm. The comb type sensors were also designed in a 2 × 2 sensor array, where sizes of individual elements were again 1 × 1, 2 × 2 and 3 × 3 cm (Figure 2). Spacing between the comb type sensor electrodes (fingers) is 0.4 mm and the electrodes themselves are 0.6 mm wide. The sensor designs are standard flat plate capacitor and comb type sensors. Width and electrode spacing was chosen as a fraction of 1 mm (0.4 mm for spacing, 0.6 mm width of electrode). Spacing and electrode width are constant for all sensors sizes, the only difference being in overall sensing area size and the number of electrodes per sensor. Flat plate sensors were perforated in such a way that the effective electrode area is 23.55% of the non-perforated plate. The average measured thickness of printed electrodes is 10 μm for all printed sensors. The FEM model was developed only after the measurements.

### 2.2. Printing of Sensors

After the sensors were designed, the printing form was prepared. Next all various printing substrate materials (Table 1) were printed using SC-CRSN2442 SunTronic 280 Thermal Drying Silver Conductive Ink. 120 L/cm high-modulus monofilament polyester plain weave mesh was applied for printing with a semi-automatic screen printer. Prints were sintered in an IR tunnel at 150 °C in 5 separate 45 s passes through the tunnel (total time 225 s) resulting in conductive ink resistance of 0.080–0.100 Ω/□. Table 2 lists substrate grammage, Vimax and PackPro are light papers and M-Liner is a cardboard substrate.

### 2.3. Analysis of Sensors Response

All printed sensors were tested in the humidity chamber at constant temperature of 23 °C and varying relative humidity level from 35% to 80%. Sensor evaluation over temperature was performed separately. Respective capacitances have been measured with the impedance measuring method at 1 kHz and 1 V_pp_. We have also devised a switching circuit for the purpose of measurement automation. This has allowed us to simultaneously track up to 16 independent sensors for specified time and sampling period (sampling time). Duration of each measurement was around 10 h (allowing extra time for humidity chamber stabilization) and the sampling period was 30 s.

**Figure 3.**
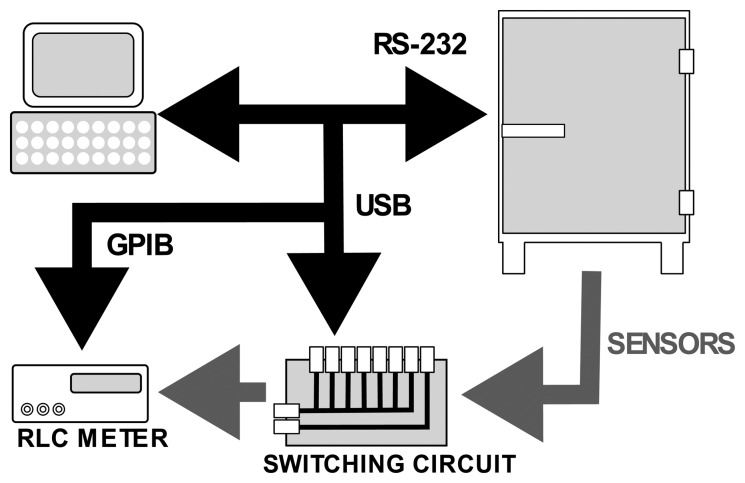
Evaluation setup.

Capacitance measurement was done through a HP RCL-meter with an impedance method at 1 kHz and 1 V_pp_ applied voltage to the sensors. The frequency of 1 kHz was chosen because of the future applications, where sensors would be integrated into a measuring platform which measures capacitance with the impedance method at 1 kHz. The 1 V_pp_ voltage was chosen arbitrarily. Quantities of interest were capacitance and conductance of each sensor. Measurements were automated via the LabView software package as shown in Figure 3.

The humidity generating device is a large humidity chamber (KK-2310 CH), produced by a local measuring equipment manufacturer. The chamber can be pre-programmed and the program is limited to 10 steps (10 changes in RH, temperature or both). The chamber also has rubber seals at the sides, through which sensor wires were pulled out so that the effect on the internal atmosphere of the chamber is minimal or almost none.

An important evaluation was done in a cyclic manner, where relative humidity was changed from 35% RH to 80% and back to 40% RH with 10% RH step and with step duration of one hour and also a reverse humidity cycle was done starting at 80% RH. This was done to test the sensor hysteresis and repeatability. To test repeatability further, the last series was done three days after the first in the exactly same manner as the first series. Total duration of each series was approximately ten hours. Also one extra series was carried out to determine the effect of temperature on capacitance, where temperature was changed from 15 °C to 40 °C in 5 °C step with duration on one hour at a constant relative humidity level of 50% RH.

## 3. Results and Discussion

The objective of the testing conducted in the humidity chamber was to identify the optimal sensor design, in size (economically) and in sensor response, for future use and further research and development.

**Figure 4.**
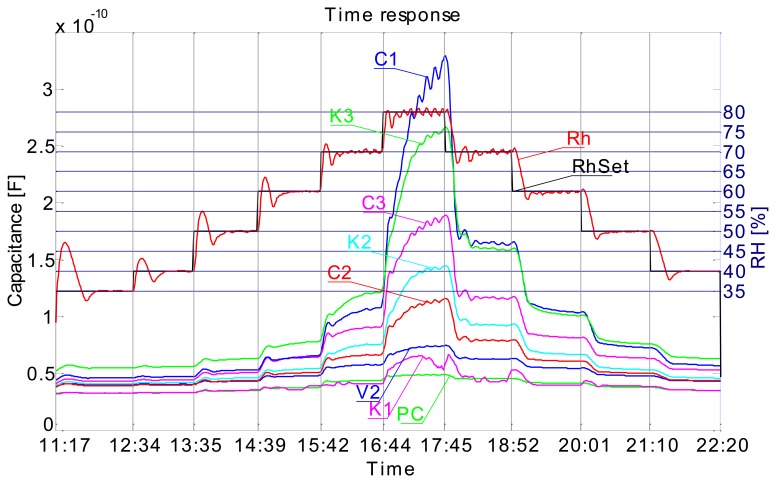
Time response of comb sensors on different material (C1–C3 recycled paper, K1–K3 cardboard, PC—poly-carbonate 2 × 2 cm, V2—food packaging paper, RhSet—humidity chamber set value, Rh—humidity chamber measured value); Sensors suffix: 1—1 × 1 cm, 2—2 × 2 cm, 3—3 × 3 cm.

**Figure 5.**
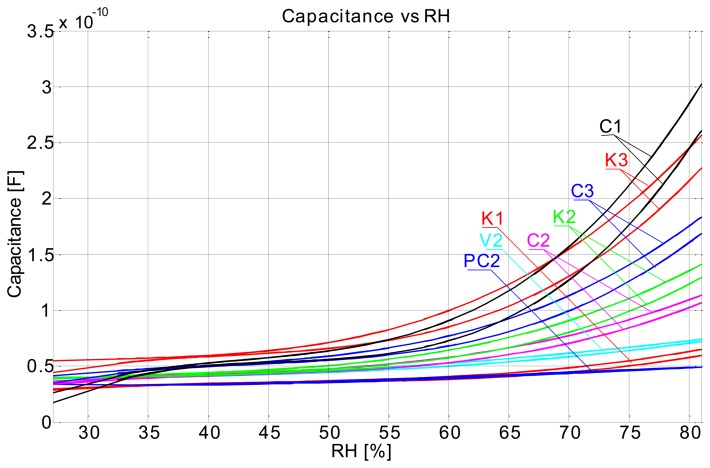
Capacitance *vs.* RH plot.

Figure 4 shows the time response of sensors and how the measurements have been carried out. In this measurement the relative humidity level (RH) in the chamber was varied from 35% RH to 80% RH and then back to 40% RH in 10% RH steps, where the duration of each step was 1 h long. In Figure 4 we can observe the time response curves of sensors and how they change when the relative humidity level is changed. We can also observe that the transient period is completed sooner (flattening of the curve after the change) at lower relative humidity levels. It is obvious that all sensors have similar responses and that the response is significantly higher at higher RH values (from 60% RH). It is also evident that the response includes a hysteresis. This is immediately seen if one, for example, compares C1 sensor's response at 70% RH prior and after the 80% RH step. Prior to the 80% RH step the C1 sensor was relatively dry and after the 80% RH step it was very moist. It is seen that a difference in capacitance is significant at same RH level prior and after saturating it with moisture. But such is the case also if RH level is significantly increased (e.g., 10%) and lowered back again at any range of relative humidity, this is clearly seen in Figure 5.

The 1 h steps and maximum RH level of 80% choice was based on our previous preliminary measurements. In preliminary measurements we have used only flat plate capacitor sensors (1st generation) to define the range of RH and time required to complete the transient phase of sensors. This measurement was conducted in such a manner to comply with the international standard ISO-187 “Paper, board and pulps—Standard atmosphere for conditioning and testing and procedure for monitoring the atmosphere and conditioning of samples”. Thus the samples were left in the humidity chamber for 24 h for every change of relative humidity at constant temperature of 23 °C (as per standard). What we have learned from this data is, that the transient phase of sensors is completed in about 45 min and that a maximum RH level is at RH 80%. Looking at the graph in Figure 4 one could argue that a transient phase is not complete, especially looking at the RH 80% (maximum limit level) response where it is clearly seen that a transient is probably just finishing. But at other RH levels we can see that a transient phase has indeed finished in about 45 min by the flattening of the curve of each sensor. Unfortunately we cannot control humidity chamber directly via the computer and we could not prolong the time step at 80% RH from 1 h to maybe 2 h or more. We decided to use the 1 h time step since most transient phases (at lower RH levels) complete in this time. Figure 5 was obtained by polynomial curve fitting (3rd order polynomial) of the data, where the data was arranged in such a manner that the *x*-axis represents all of the unique measured RH levels and respective *y*-axis sensor responses (capacitance). One curve is obtained as the RH level was increasing and the other when RH level was falling.

**Figure 6.**
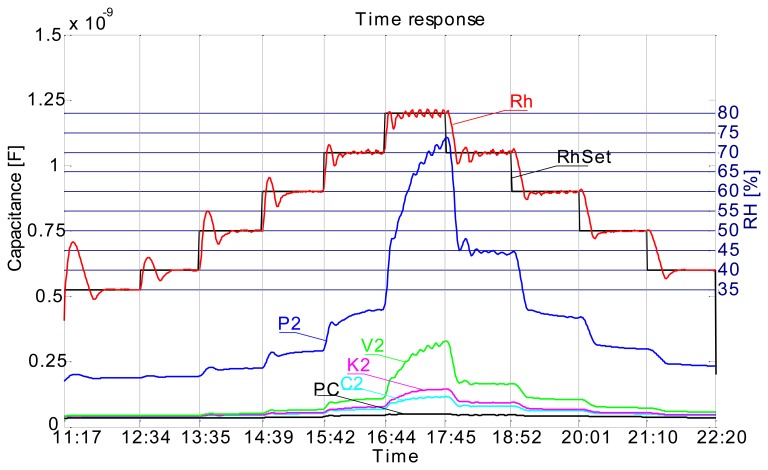
Comparison of the same dimension capacitors (2 × 2 cm); P2—perforated flat plate capacitor (recycled paper), C2—comb capacitor (recycled paper), K2—comb capacitor (cardboard), PC—comb capacitor (poly-carbonate), V2—comb capacitor (food packaging paper), RhSet—humidity chamber set value, Rh—humidity chamber measured value).

**Figure 7.**
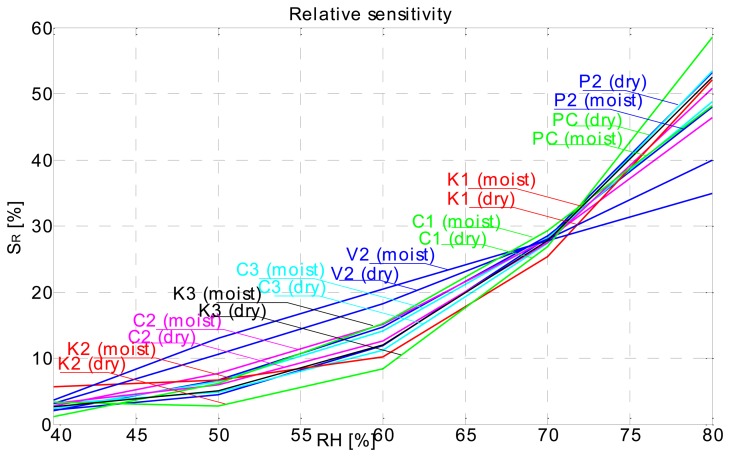
Relative sensitivity plot.

Our conclusion in this case is that the sensor response is not so much a function of the substrate, but more a function of the conductive ink. For final conclusions the electrical properties of the conductive ink as a function of relative humidity have to be more thoroughly researched, this will be the main focus of our future work. Regarding the perforated flat plate sensors' performance, we can say that the response is in no way different from the rest of the sensors. Greater absolute capacitance values have been obtained from the 2 × 2 cm perforated flat plate capacitor (Figure 6), due to the much larger surface area of the electrodes. Relative sensitivity of this kind of sensor is almost identical with other types of sensors (Figure 7).

The rise and fall times of sensors are too rapid for what would be expected from a cellulose-based material. Furthermore there is no significant difference in sensor response, in relative terms, with cellulose-based substrates and the ones based on synthetic substrates (PC–foil), which is confirmed in Figure 7, which is a relative sensitivity plot calculated by Expression (1):

(1) $$S_{R}=\frac{\Delta C(\Delta Rh)}{C_{\max}-C_{\min}}\cdot 100\%$$

Our final conclusion about the optimum sensor design for the future use is to continue our work with the smallest (1 × 1 cm) comb sensor and alternatively to try with even smaller sensor dimensions.

## 3.1. Conductance of Sensors

**Figure 8.**
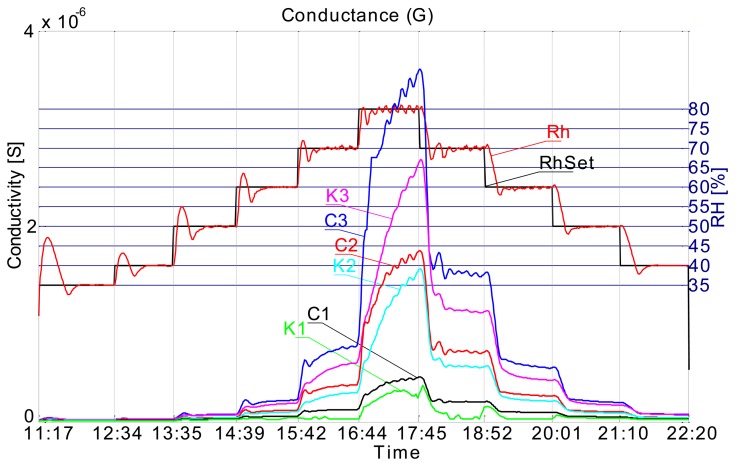
Conductance of comb type sensors on recycled paper and cardboard.

The measuring setup we used has allowed us to also track the respective conductance of individual capacitors (sensors) to evaluate ohmic loss in different substrates. Conductance is parallel to the capacitance in a capacitor. Conductance data plots show a similar trend as capacitance plots (Figure 8). Conductivity of the medium is rising as the relative humidity level rises.

**Figure 9.**
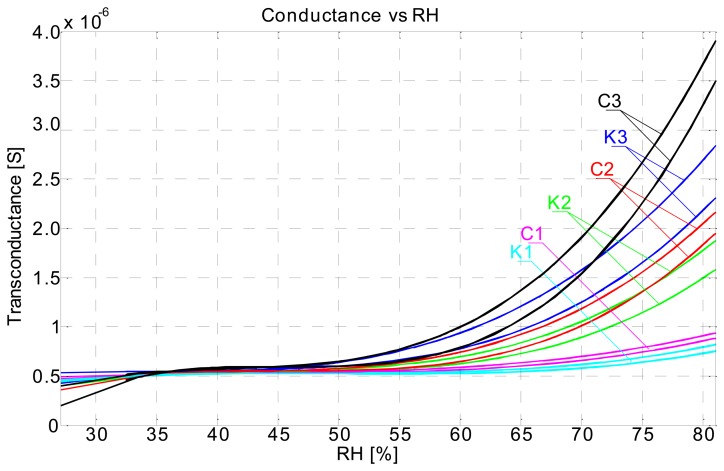
Conductance *vs* relative humidity plot.

Conductance also includes a hysteresis response; moist sensors have higher conductivity (lower resistance) than when dry (Figure 9).

**Figure 10.**
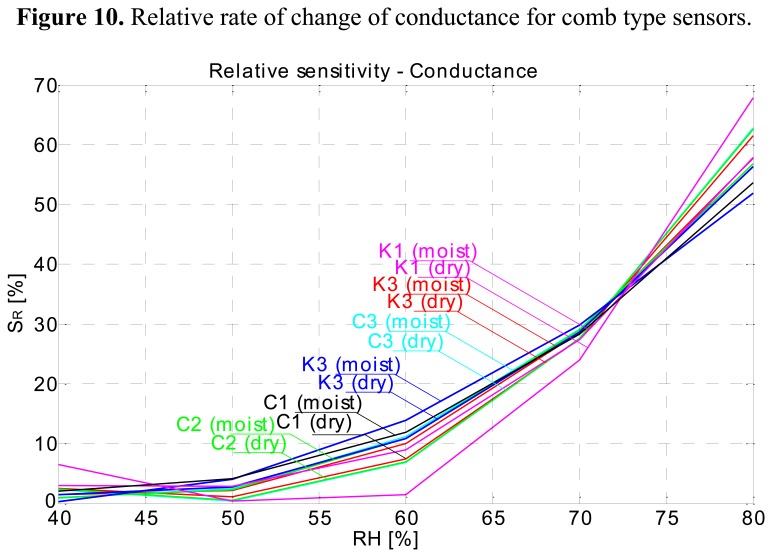
Relative rate of change of conductance for comb type sensors.

Relative rate of change (relative sensitivity) shows no significant difference between the two materials (Figure 10).

Again we found that the conductance sensor response is not a function of the substrate, when one considers the relative terms (relative sensitivity).

### 3.2. Sensor Impedance

From the measurement data (capacitance and conductance) we are able to derive the capacitor (or sensor) impedance using the Expression (2):

(2) $$\begin{array}{ccc} R_{x}=\frac{1}{G_{x}}; & Z=\frac{R_{x}}{1+j\omega R_{x}C_{x}}; & \omega =2\pi \;f,f=1\;kHz \end{array}$$

**Figure 11.**
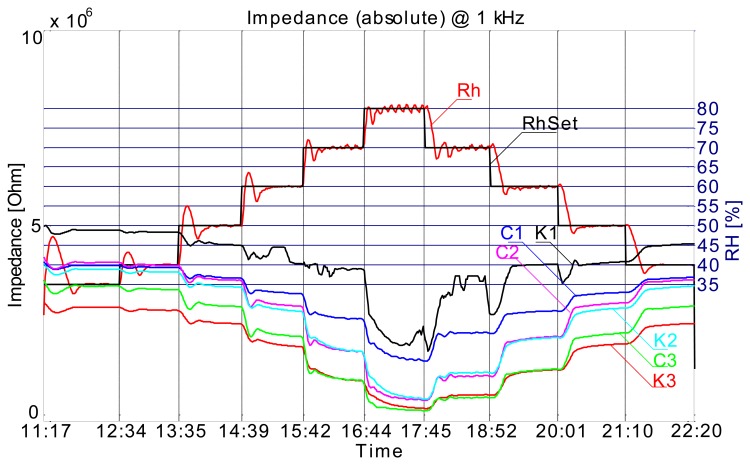
Impedance of comb type sensors on recycled paper and cardboard.

Figure 11 shows the impedance plot for comb type sensors. While there is a slight difference in absolute values between the two materials, we argue that the biggest difference is due to the dimensions of the respective sensors. We can easily see that the sensors with the same dimensions have almost the same response (C1–K1, C2–K2, C3–K4).

**Figure 12.**
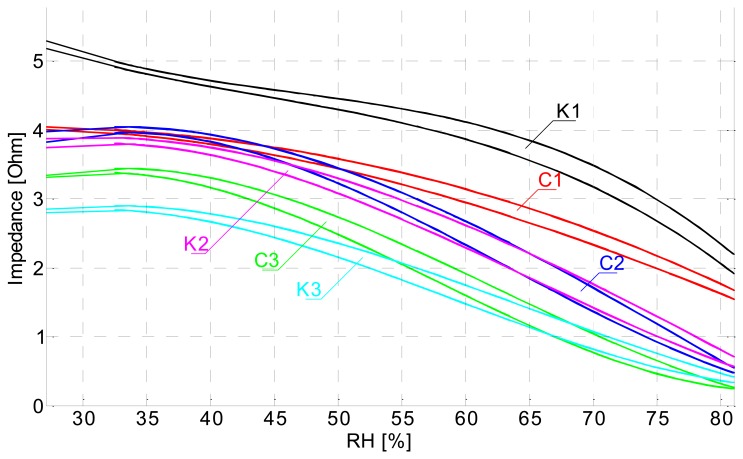
Impedance *vs.* RH plot of comb type sensors on recycled paper and cardboard.

In Figure 12 we can observe the hysteresis and non-linear response of comb type sensors. It is also very obvious that the sensors with same dimensions share almost the same response, regardless of the substrate (e.g., C2–K2).

### 3.3. Repeatability of Sensors

**Figure 13.**
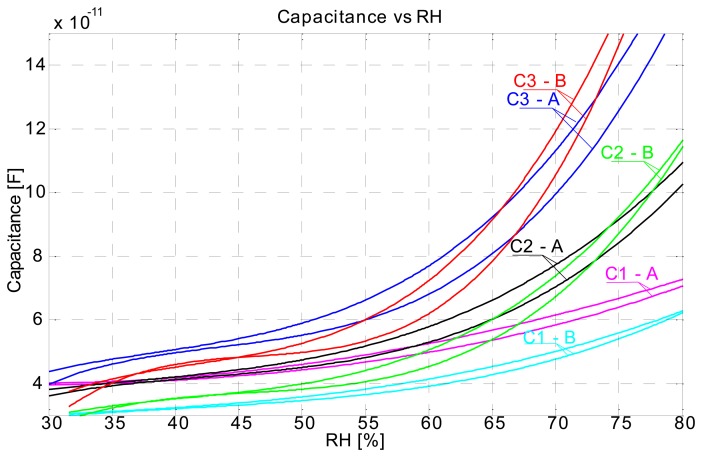
Capacitance *vs.* RH plot for two measurement series (A and B).

Repeatability of sensors is poor. Figure 13 shows a capacitance *vs* RH plot for two different measurement series. The A series is the series from Figure 4, and B series data is from the measurement inverse to the A series, where we have started from 80% RH to 35% and back to 70%. We observe from Figure 13 that the respective sensor response is different from series A to series B. We had expected that the response in the B series would be somewhat higher, due to the high RH starting level and moistening of the sensors, which this was not the case. Also extreme difference in response is observed with the C1 sensor, where responses A and B do not overlap at all.

### 3.4. Temperature Analyses

**Figure 14.**
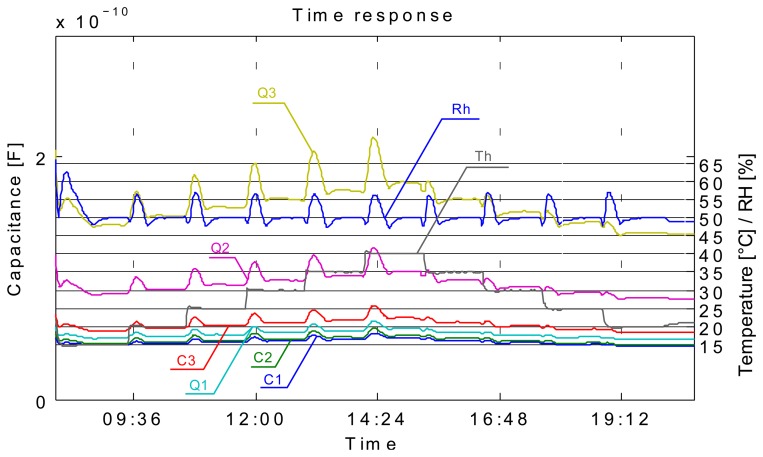
Printed sensor response with temperature.

One measurement series was carried out to determine the effect of temperature on the capacitance of the sensors. As shown in Figure 14, the impact of temperature is not very relevant, but should be taken into account in any real application. It is interesting to note how in Figure 14 spikes in the capacitance response when the temperature changes are clearly seen, but after a few minutes the capacitance returns almost back to the original level. These spikes seen in Figure 14 originate from the change in relative humidity (Rh–curve) in the chamber as the temperature (Th–curve) is suddenly changed. After a while the humidity chamber is able to compensate for change in temperature and the relative humidity is restored to the 50% RH level. It is obvious that a sensor response is almost identical to the Rh–curve, which is another evidence of a fast sensor response. Other interesting fact is that sensor response is not very temperature dependant, as is clearly seen from the Figure 14. The difference in capacitance at the maximum temperature of 40 °C and minimum temperature of 20 °C is very small. A study of exact sensor dynamics will be part of our future work.

### 3.5. Sensory Tag Application

The humidity sensor presented in [19] is a passive UHF RFID humidity sensor tag fabricated using inkjet technology. This paper describes the structure and operation principle of the sensor tag and discusses the method of performing humidity measurements in practice as well.

**Figure 15.**
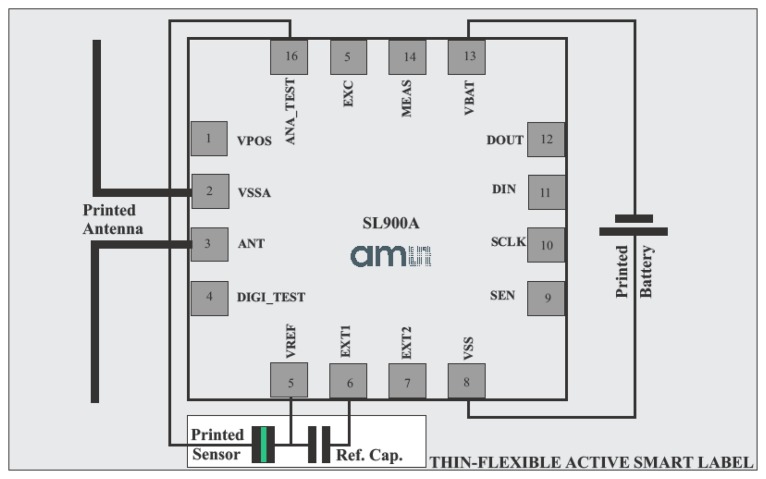
Practical-sensory tag application, based on SL900A smart label with integrated temperature sensor, real time shelf-life calculation and data logging capability, combined with external screen printed humidity sensor, and screen printed UHF antenna.

**Figure 16.**
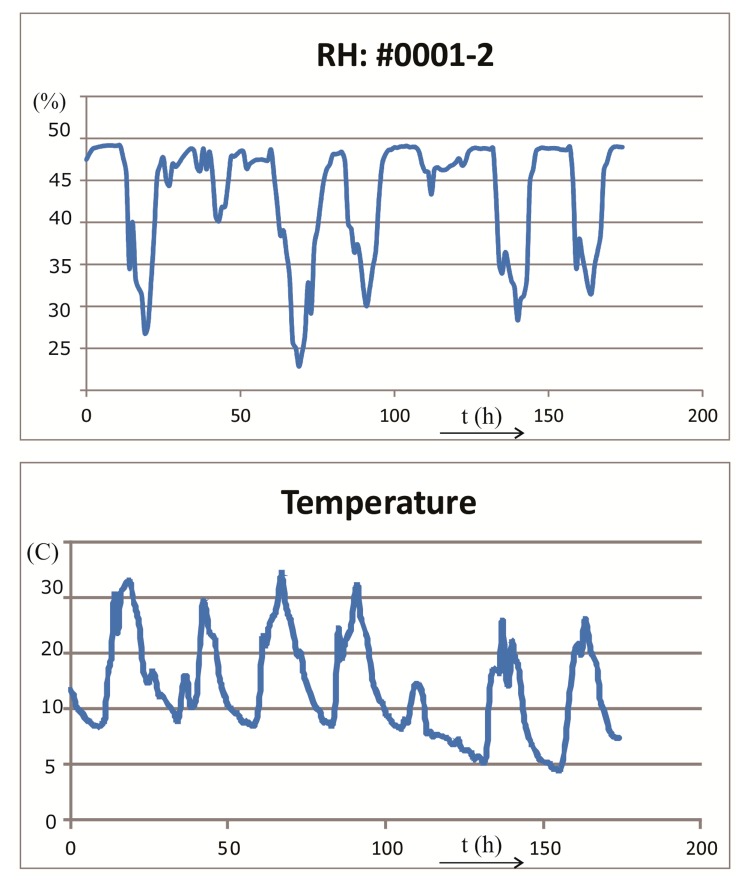
Measured relative humidity using the printed sensor and on board integrated temperature sensor.

The wireless sensor application that provides tracking capability is shown in Figure 15 and the corresponding temperature and humidity measurements are shown in Figure 16. The same screen printing technique was used for printing the humidity sensor and for the UHF antenna [2]. Reference and measured capacitive sensor are AC supplied. The SL900A is an EPC Class 3 tag chip enabling affordable RFID automatic data logging applications with sensor functions [20]. The SL900A works in semi-passive (battery-assisted) mode as well as in fully passive mode. Battery support enables data logging and burst communication range with reader. Sensor data readout is also possible in fully passive operating mode—without battery.

Data was taken once every hour (*x*-axis), for 7 days, and was stored in the SL900A's memory. The application was placed in the open air, where conditions during the day and night are clearly recognized. Sensors were exposed to direct sun in the middle of the day (measured in May 2014).

## 4. Conclusions

Screen printed capacitive humidity sensors of various dimensions and configurations have been successfully designed, fabricated and tested. Tests were conducted in a humidity chamber by varying the relative humidity levels and measuring the changes in capacitance. One of the objectives was to define an optimal sensor design (in terms of size and response) for future use. Analysis has shown that such a sensor is in fact the smallest 1 × 1 cm comb type sensor which can probably be made even smaller. Response characteristics are not in accord with our expectations. We had expected to see differences in sensor response depending on the substrate used (Figure 7), even more so between the cellulose based substrates and synthetic ones (poly-carbonate foil). Also the sensor response is too fast for a cellulose-based substrate response, where delays in the range of tens of minutes were expected. Instead, sensor rise times are very short, so much so that we can observe an overshoot in the response as the sensor is tracking the rising or falling of relative humidity level in the chamber almost by the minute (Figure 4). Sensors are more sensitive at a higher relative humidity range and we can conclude that their behaviour is generally better when sufficiently moist. Sensor response includes a hysteresis (Figure 5), which is expected. We conclude that the sensor response is very much more a function of the conductive ink than a function of the substrate. There is no significant change in performance in the case of perforated flat plate capacitor or the comb type capacitor, which was also not expected. Another troubling aspect of the sensor performance is the repeatability of the results. As seen from Figure 13 the repeatability of the sensor response is very poor. We believe we can significantly improve the response by using a different ink (e.g., carbon-based ink) and/or by coating the sensors with a polymer, which would also broaden the spectrum of applications for the sensors (e.g., chemical gas detector…). We present the successful use of the proposed screen printing technique on paper/polymer based substrates by demonstrating simple sensor node application that could be easily extended to other wireless applications.

## Figures and Tables

**Table 1.** Printing substrates and sensor labelling.

**Substrates and Sensor Labelling**	
Vimax (recycled paper)	C1 (1 × 1 cm), C2 (2 × 2 cm), C3 (3 × 3 cm); comb type
M-Liner (cardboard)	K1 (1 × 1 cm), K2 (2 × 2 cm), K3 (3 × 3 cm); comb type
PackPro (food packaging paper)	V2 (2 × 2 cm); comb type
Polycarbonate foil	PC (2 × 2 cm); comb type

**Table 2.** Substrate grammage.

**Substrates and Sensor Labelling**	
Vimax (recycled paper)	70 g/m^2^
M-Liner (cardboard)	230 g/m^2^
PackPro (food packaging paper)	50 g/m^2^
